# Adding physical activity to intensive trauma-focused treatment for post-traumatic stress disorder: results of a randomized controlled trial

**DOI:** 10.3389/fpsyg.2023.1215250

**Published:** 2023-07-20

**Authors:** Eline M. Voorendonk, Sarita A. Sanches, Marieke S. Tollenaar, Elisabeth A. Hoogendoorn, Ad de Jongh, Agnes van Minnen

**Affiliations:** ^1^Research Department, PSYTREC, Bilthoven, Netherlands; ^2^Behavioral Science Institute (BSI), Radboud University Nijmegen, Nijmegen, Netherlands; ^3^Phrenos Centre of Expertise for Severe Mental Illness, Utrecht, Netherlands; ^4^Altrecht Institute for Mental Health Care, Utrecht, Netherlands; ^5^Institute of Psychology, Department of Clinical Psychology and the Leiden Institute for Brain and Cognition, Leiden University, Leiden, Netherlands; ^6^Faculty of Science, Leiden University, Leiden, Netherlands; ^7^Academic Centre for Dentistry Amsterdam (ACTA), University of Amsterdam and VU University Amsterdam, Amsterdam, Netherlands; ^8^School of Psychology, Queen’s University, Belfast, United Kingdom; ^9^Institute of Health and Society, University of Worcester, Worcester, United Kingdom

**Keywords:** post-traumatic stress disorder, intensive trauma-focused therapy, physical activity, randomized controlled trial, clinician-administered PTSD scale, PTSD checklist for DSM-5

## Abstract

**Introduction:**

This randomized controlled trial examined the effectiveness of physical activity added to an intensive trauma-focused treatment (TFT) for post-traumatic stress disorder (PTSD) in comparison to adding non-physical control activities.

**Methods:**

A total of 119 patients with PTSD were randomly assigned to a physical activity condition (PA; *n* = 59) or a non-physical activity control condition (nPA; *n* = 60). The 8-day intensive TFT programme consisted of daily prolonged exposure, EMDR therapy, and psychoeducation, which was complemented with physical activities versus controlled mixtures of guided (creative) tasks. As a primary outcome, the change in clinician and self-reported PTSD symptoms from pre-to post-treatment and at 6 months follow-up were measured.

**Results:**

Intent-to-treat linear mixed-effects models showed no significant differences between the PA and nPA conditions on change in PTSD severity. Clinician and self-reported PTSD symptoms significantly decreased for both conditions, with large effect sizes (e.g., CAPS-5 *d_pre-post_* = 2.28). At post-treatment, 80.0% in the PA, and 82.7% in the nPA condition no longer met the diagnostic criteria for PTSD. Regarding the loss of Complex PTSD diagnoses this was 92.5% and 95.0%, respectively.

**Conclusion:**

Either with additional physical or non-physical activities, intensive TFT is very effective for the treatment of (Complex) PTSD, as reflected by large effect sizes and loss of diagnostic status in both groups.

**Clinical trial registration:**

Trialregister.nl Identifier: Trial NL9120.

## 1. Introduction

In the last decade, intensive trauma-focused treatments (TFT) for individuals with post-traumatic stress disorder (PTSD) have increasingly gained attention and have been found to be effective in reducing PTSD symptoms within a short period of time (Ehlers et al., 2014; Foa et al., 2018; Hoppen et al., 2023). These novel programmes often integrate physical activities into the treatment format (Van Woudenberg et al., 2018; Zalta et al., 2018; Zepeda Méndez et al., 2018; Voorendonk et al., 2020; Brynhildsvoll Auren et al., 2022). Adding physical activity to TFT is thought to work as a potential augmentation strategy, based on research showing that usual care (including TFT) for PTSD, augmented with physical activity sessions, showed a significantly stronger decrease in PTSD symptoms than usual care alone (Rosenbaum et al., 2015a). Besides, evidence suggests that exercise as a stand-alone treatment for PTSD is capable of reducing PTSD symptoms (Fetzner and Asmundson, 2015; LeBouthillier et al., 2016; Goldstein et al., 2018; Whitworth et al., 2019a; Hall et al., 2020).

While meta-analyses support these favorable but preliminary results (Rosenbaum et al., 2015b; Björkman and Ekblom, 2021; Davis et al., 2021; Ramos-Sanchez et al., 2021), the evidence for an augmentation effect of physical activity on TFT remains inconclusive owing to several study limitations. Most studies included in meta-analyses did not include non-physical activity control conditions (Fetzner and Asmundson, 2015; Powers et al., 2015; Rosenbaum et al., 2015a; LeBouthillier et al., 2016; Goldstein et al., 2018; Hall et al., 2020; Nordbrandt et al., 2020). Hence, it is conceivable that non-specific effects of physical activity sessions, such as receiving (more) attention or social support, may have been responsible for the stronger decrease in PTSD symptoms. Additionally, the studies included in the meta-analyses on this topic were either pilot studies with small sample sizes and homogeneous specific samples (e.g., veterans), or lacked follow-up measurements (Powers et al., 2015; Rosenbaum et al., 2015a; Goldstein et al., 2018; Whitworth et al., 2019a,b; Hall et al., 2020; Nordbrandt et al., 2020). With homogeneous samples of veterans with war-related trauma or predominantly males it remains unclear whether these beneficial results can be generalized to patients with PTSD who have been exposed to other types of trauma or women with PTSD, who have an approximately twofold higher risk of developing PTSD after trauma exposure and show higher lifetime prevalence rates of PTSD than men (De Vries and Olff, 2009). More importantly, the unique contribution of physical activity has never been investigated in the context of intensive TFT (Voorendonk et al., 2019), which is highly relevant, given its effectiveness and proliferation (Ragsdale et al., 2020; Sciarrino et al., 2020; Hoppen et al., 2023). Outside the context of intensive TFT, a recent controlled study found that the addition of brief aerobic exercise after prolonged exposure therapy sessions was more effective in reducing PTSD symptom severity than an active control condition consisting of passive stretching (Bryant et al., 2022). However, this significant beneficial effect on PTSD symptom severity was not observed post-treatment, but only at the 6 months follow-up. These 6 months after treatment, however, were uncontrolled; thus, it is unknown what participants did in terms of trauma-focused treatment or physical activity, making it difficult to draw strong conclusions about physical activity as a potential augmentation strategy for prolonged exposure therapy in patients with PTSD.

The purpose of this randomized controlled trial was to investigate the effectiveness of physical activity added to an intensive TFT programme for patients with PTSD, incorporating a non-physical activity control condition. We hypothesized that patients in the physical activity condition (PA) would show a significantly greater decrease in PTSD symptoms from pre-to post-treatment than those in the non-physical activity control condition (nPA) (Voorendonk et al., 2022). We further hypothesized that this difference would endure at 6 months follow-up and would result in a higher loss of (Complex) PTSD diagnostic status. Secondary outcome measures included lifestyle variables and assessment of other psychiatric and somatic problems. Finally, we examined whether treatment preference influences treatment effectiveness.

## 2. Materials and methods

### 2.1. Participants and design

This single-center, randomized controlled trial with two conditions was conducted in the Netherlands between December 2020 and March 2022. The study protocol and detailed procedures, which have been published elsewhere (Voorendonk et al., 2022), were approved by the Medical Ethics Review Committee of the VU University Medical Centre (VUmc: IRB00002991, FWA0001 7598) on May 15, 2020 (reference number: 2019.574 – NL70812.029.19). After the study procedures had been fully explained and after at least a week of consideration, all participants provided written informed consent. The trial followed the Consolidated Standards of Reporting Trials (CONSORT) reporting guidelines and was listed on trialregister.nl (Trial NL9120).

All recruited patients were regular referrals to PSYTREC, a clinic specializing in the intensive treatment of PTSD. The inclusion criteria were as follows: (1) PTSD diagnosis based on the Clinician-Administered PTSD Scale (CAPS-5; Boeschoten et al., 2018), (2) age > 18 years, and (3) sufficient knowledge of the Dutch language. The exclusion criteria were: (1) suicide attempt < three months prior to treatment and (2) medically unfit, i.e., not being able to walk for at least 30 minutes. Recruitment continued until 120 patients provided onsent.

A web-based randomization program (www.randomizer.org) was used to assign 120 participants to the two conditions (1:1) using block randomization with 20 blocks of six participants.

### 2.2. Intervention

All participants received an 8-day intervention over a time span of 2 weeks (2 × 4 consecutive days with 3 days pause in between) consisting of a combination of daily: an individual prolonged exposure (PE) session in the morning, an eye movement desensitization and reprocessing (EMDR) therapy session in the afternoon (90 min each), and brief group psychoeducation during the day and in the evening. All sessions followed protocols and were provided by therapists with a master’s degree in clinical psychology who were trained in PE and EMDR therapy (Voorendonk et al., 2020, 2022). In addition, each day participants engaged in four daily sessions (90 min each) of physical activities (physical activity condition; PA) or a controlled mix of guided tasks that did not include physical activity (non-physical activity control condition; nPA). These activities were performed in a group that consisted exclusively of people diagnosed with PTSD according to the CAPS-5 and followed a predefined protocol. The first four days were delivered face-to-face (inpatient) and the last four days were delivered remotely. Of importance, all participants received the same duration and frequency of the treatment components PE, EMDR therapy and psychoeducation. Only the augmentation part differed: PA or nPA interventions. The timing of the PA or nPA sessions in relation to the PE and EMDR sessions (before, after or both) varied per person and per day for pragmatic reasons. Participants received equal proportions of all sequence combinations in both conditions. For additional details see (Voorendonk et al., 2022).

#### 2.2.1. PA condition

Physical activity consisted of a prescribed combination of aerobic and resistance training performed at a moderate intensity. Participants followed a protocol with one specific activity per session and did not have the option to engage in various activities during the same session. For the first four days, the day started and ended with a 60 min outside walking session, in which participants were instructed and supervised to walk on a tempo that made them reach their moderate intensity level based on their heart rate and perceived exertion. The other two daily sessions, based on the protocol, entailed aerobic group games (soccer, softball, basketball, and ultimate frisbee), mountain biking, boxing, ergometer, or resistance training. On average, during the first week, around 62.5% of the sessions were focused on aerobic cardiovascular training, 25% were competition/games, and 12.5% were resistance training sessions. All of these activities were performed outside in a natural area around the clinic or in an indoor fitness room. The online physical activity sessions during the last four days, were delivered through Zoom Meetings. They consisted of individual walking sessions at the start and end of the day, daily live-lessons wherein the participants were required to be visible on screen (e.g., boxing, and dancing), or performing other physical activities at home (e.g., biking). On average, during the second week, 80% of the sessions were focused on aerobic cardiovascular training, and the remaining 20% was dedicated to resistance training. All online sessions started and ended with group Zoom meetings. Overall, 50% of the physical activities were conducted in person (week 1) and 50% online (week 2). Furthermore, 100% of the physical activities during the first week and around 25% of the activities during the second week were completed in a group format. Moreover, 85% of the physical activities during both weeks were performed outside in a natural environment. No therapeutic interventions, such as psychomotor therapy, were administered during any of the activities.

#### 2.2.2. nPA condition

The controlled and prescribed mix of guided (creative) tasks consisted of activities that did not involve physical activity and could be performed at a low heart rate. All tasks were performed while seated and consisted of craft activities using different materials (e.g., paint, clay, crochet), accompanied by assignments and themes, interactive board games, or reading. During the first four days of treatment, the controlled tasks were performed at our indoor room or outside in a natural area surrounding the clinic. During the last four days online, the activities were performed individually at home with craft materials and assignments or live lessons in a group format (e.g., online games). All online sessions started and ended with group Zoom meetings. Overall, similar to the PA condition, 50% of the activities were conducted in person (week 1) and 50% online (week 2). Furthermore, similar to the PA condition, 100% of the activities during the first week and around 25% of the activities during the second week were completed in a group format. In contrast to the PA condition, 10% of the activities during both weeks were performed outside. No therapeutic interventions were administered during any of the tasks.

#### 2.2.3. Compliance and fidelity monitoring

To assess compliance and monitor protocol fidelity, the heart rate of each participant was recorded during every physical or non-physical activity session with individual Fitbit Charge 4 heart rate monitors. Additionally, a subjective perception rating scale was completed at the end of each session (Borg’s Rating Sale of Perceived Exertion [RPE]; Borg, 1998). These measures monitored the overall attendance rates and times and whether the intended moderate intensity in the PA condition (60–70% of Maximum Heart Rate [MHR] / RPE ≥ 12) and inactivity in the nPA condition were achieved. Compliance with the PA and nPA protocols was supervised by instructors with a degree in human movement sciences and trained to work with people with PTSD.

### 2.3. Outcome measures

The primary outcome measured over time was PTSD symptom severity, assessed using the Clinician-Administered PTSD Scale (CAPS-5; Boeschoten et al., 2018) and the self-reported PTSD Checklist for DSM-5 (PCL-5; Boeschoten et al., 2014), both ranging from 0 to 80. The CAPS-5 was also used to assess (loss of) PTSD diagnosis. The CAPS-5 was conducted by psychologists with a master’s degree in clinical psychology who were appropriately trained. The assessors could not be blinded for treatment conditions due to pragmatic reasons.

The secondary outcomes evaluated included sleep problems (Insomnia Severity Index [ISI]; score range 0–28), depressive symptoms (Quick Inventory of Depressive Symptomatology Self-Report [QIDS-SR]; score range 0–27), emotion regulation problems (Difficulties in Emotion Regulation Scale [DERS]; score range 36–180), dissociative symptoms (Dissociative Experiences Scale-II [DES-II]; score range 0–100), anxiety sensitivity (Anxiety Sensitivity Index [ASI]; score range 0–64), quality of life (Manchester Short Assessment of quality of life [MANSA]; score range 12–84), physical activity level (International Physical Activity Questionnaire Short Form [IPAQ-SF]), general somatic complaints (somatization subscale of Symptom Check List-90 [SCL-90]; score range 12–60), and Complex PTSD symptoms and diagnosis (CPTSD; International Trauma Questionnaire [ITQ]; score range 0–48) (see the study protocol Voorendonk et al., 2022 for detailed descriptions).

All outcomes were assessed at pre-treatment, post-treatment, and 6 months follow-up. In addition to determining the participants’ sex at birth (female/male), gender identity was ascertained using an additional question (female/male/other). To determine ethnicity, the country of birth of the participants and their parents was assessed.

### 2.4. Statistical analysis

Based on previous studies (Powers et al., 2015; Rosenbaum et al., 2015a,b; Goldstein et al., 2018; Whitworth et al., 2019a; Hall et al., 2020; Ramos-Sanchez et al., 2021), a moderate effect size was expected. Assuming a moderate effect size, the power analysis showed that a minimum sample size of 86 participants was required to detect a significant difference between the conditions in our primary analyses, with a power of 80% (*α* = 0.05, *f* = 0.25). Considering the possibility of dropout, technical measurement failure and missing data (Rosenbaum et al., 2014, 2015a), 120 participants were included in the study. For both the primary (CAPS-5 and PCL-5) and secondary outcome measures, we performed intent-to-treat linear mixed-effects regression analyses with repeated measures. The final models were all Multilevel Models of Change (MMC) with random intercepts and random time slopes across persons and fixed effects of time, condition (PA vs. nPA), and the time x condition interaction. Time was treated categorically. Statistical analyses were conducted using SPSS version 27.0, and the nlme package in R version 4.1.3 (MMC analyses). Hypothesis tests were 2-sided with an alpha value of 0.05.

## 3. Results

**Figure 1:**
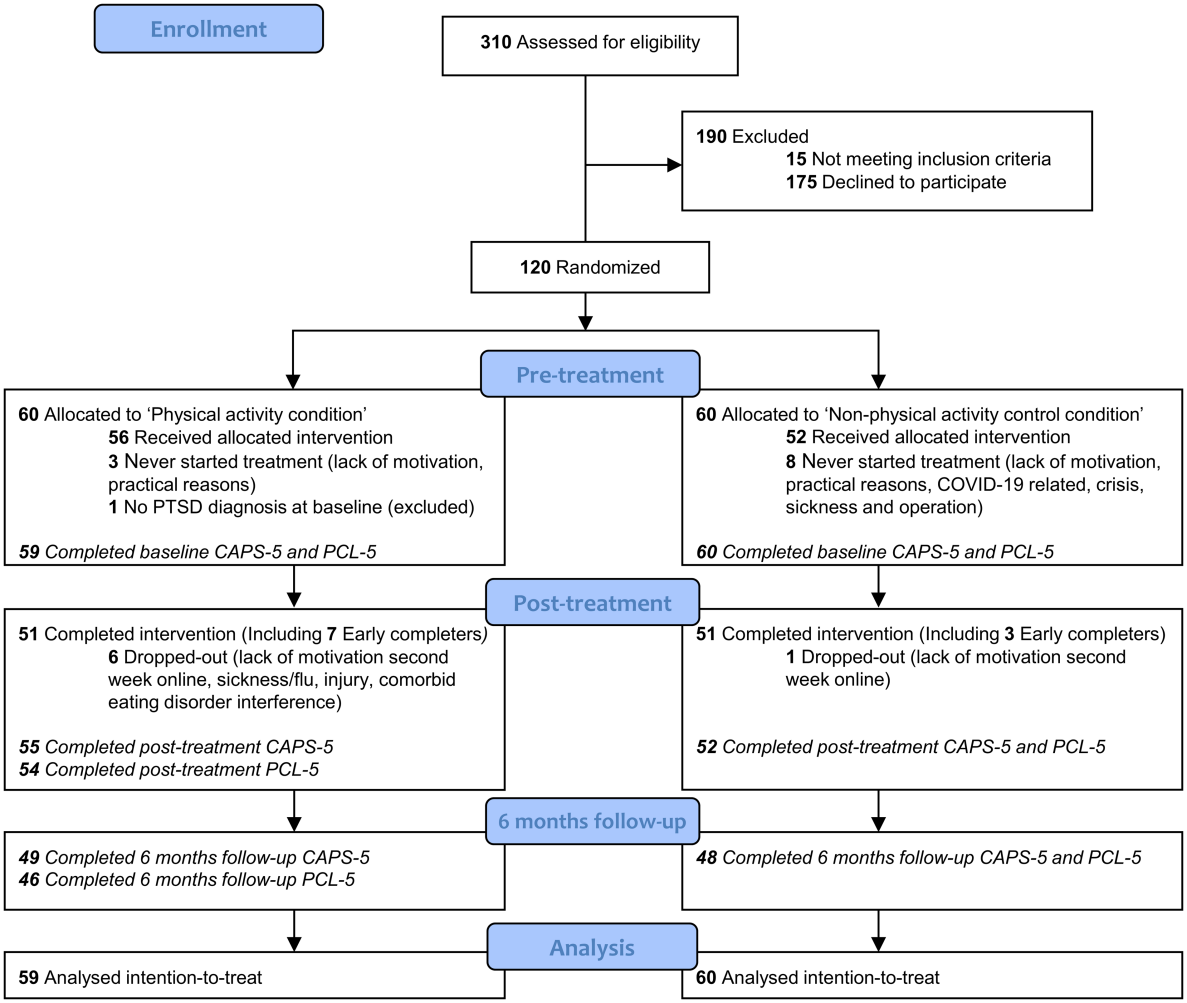
CONSORT participant flow. CAPS, Clinician-Administered PTSD Scale; PCL-5, PTSD Checklist for DSM-5. Early completers = participants who ended treatment successfully earlier than the eight treatment days (in 4 to 5 days).

A total of 310 patients were screened for eligibility, of which 120 were included and randomly assigned to either the PA condition (*n* = 60) or the nPA condition (*n* = 60). One participant in the PA condition was excluded after randomization because they did not fulfil the diagnostic criteria for PTSD at baseline (Figure 1; see Supplementary Results for sensitivity analysis with participant included). The conditions did not significantly differ with respect to baseline characteristics (Table 1). The 119 participants (84.9% female; mean age = 37.50, *SD* = 11.57, range 19–74) showed high rates of sexual (86.6%) and physical abuse (93.3%) and elevated suicide risk (66.4%). Seventy-six percent of the participants had Dutch ethnicity, while the rest had Moroccan (3%), Turkish (2%), Surinamese (3%), or other (16%) backgrounds. The majority of the participants were moderately or highly physically active (32.8% and 40.3% respectively; IPAQ-SF), had an average BMI of 25.75 (*SD* = 5.71), and reported low subjective physical fitness scores (*M* = 3.76, *SD* = 1.52, score range 0–10) at baseline. Moreover, the conditions did not differ in the number of participants who never started treatment or ‘early completers’, but significantly more dropout occurred in the PA condition (χ^2^[1] = 3.89, *p* = 0.049; 10.2% vs. 1.7%; Figure 1).

**Table 1:** Baseline characteristics of the physical activity (PA) and non-physical activity control condition (nPA).

	PA (*n* = 59)	nPA (*n* = 60)	*t* or *χ*^2^ value	*p*-value
Mean age (*SD*)	37.58 (12.51)	37.42 (10.67)	0.08	0.940
Sex (% female)^a^	51 (86.4%)	50 (83.3%)	0.22	0.636
Mean CAPS-5 score (*SD*)	43.59 (6.33)	42.77 (5.79)	0.74	0.459
Complex PTSD diagnosis^b^	40 (67.8%)	40 (66.7%)	0.23	0.633
Traumatic experiences
Sexual abuse	50 (84.7%)	53 (88.3%)	0.33	0.566
Physical abuse^c^	58 (98.3%)	53 (88.3%)	4.72	0.030
Natural disasters and severe accidents	38 (64.4%)	44 (73.3%)	1.11	0.293
Comorbidity
Mood disorders	40 (67.8%)	33 (55.0%)	2.05	0.152
Anxiety disorders	28 (47.5%)	24 (40.0%)	0.67	0.412
Suicide risk
High	9 (15.3%)	11 (18.3%)	0.20	0.653
Moderate	9 (15.3%)	8 (13.3%)	0.09	0.765
Low	19 (32.2%)	23 (38.3%)	0.49	0.484
Mean subjective physical fitness (0–10 scale)	3.71 (1.38)	3.80 (1.66)	0.32	0.753
Mean BMI^d^ (*SD*)	25.53 (5.73)	25.96 (5.72)	0.40	0.689

a One participant in the nPA condition answered “other” to the gender identity question.

b Assessed using the International Trauma Questionnaire (ITQ).

c Not significant with Bonferroni correction (α = 0.004) or Fisher’s exact test (*p* = 0.061).

d Body Mass Index (BMI) = weight(kg)/(length(m)^2^).*Note*. CAPS, Clinician-Administered PTSD Scale.

### 3.1. Manipulation check: compliance and fidelity monitoring

Due to practical reasons of preparation for the activity sessions, gatherings, and explanations, the mean duration of each timeslot was 60.78 (*SD* = 15.29) minutes in the PA condition and 61.59 (*SD* = 3.08) minutes in the nPA condition (*t*[100] = −0.37, *p* = 0.713) instead of the aimed 90 minutes. During these sessions, on average, 17.94 (*SD* = 8.69) minutes of moderate intensity activity per session was performed in the PA condition, versus 0.54 (*SD* = 0.80) minutes in the nPA condition (*t*[56.01] = 14.92, *p* < 0.001, *d* = 2.77). The mean HR and RPE scores per session were significantly higher in the PA condition (HR: *M* = 107.83, *SD* = 7.99; RPE: *M =* 11.53, *SD* = 0.82) than in the nPA condition (HR: *M* = 81.97, *SD* = 7.71; *t*[106] = 17.10, *p* < 0.001, *d* = 3.29; RPE: *M =* 7.05, *SD* = 1.08; *t*[106] = 24.46, *p* < 0.001, *d* = 4.71). These findings indicate a sufficient contrast in physical activity intensity between the two conditions.

### 3.2. Analysis of primary outcome measures

**Figure 2:**
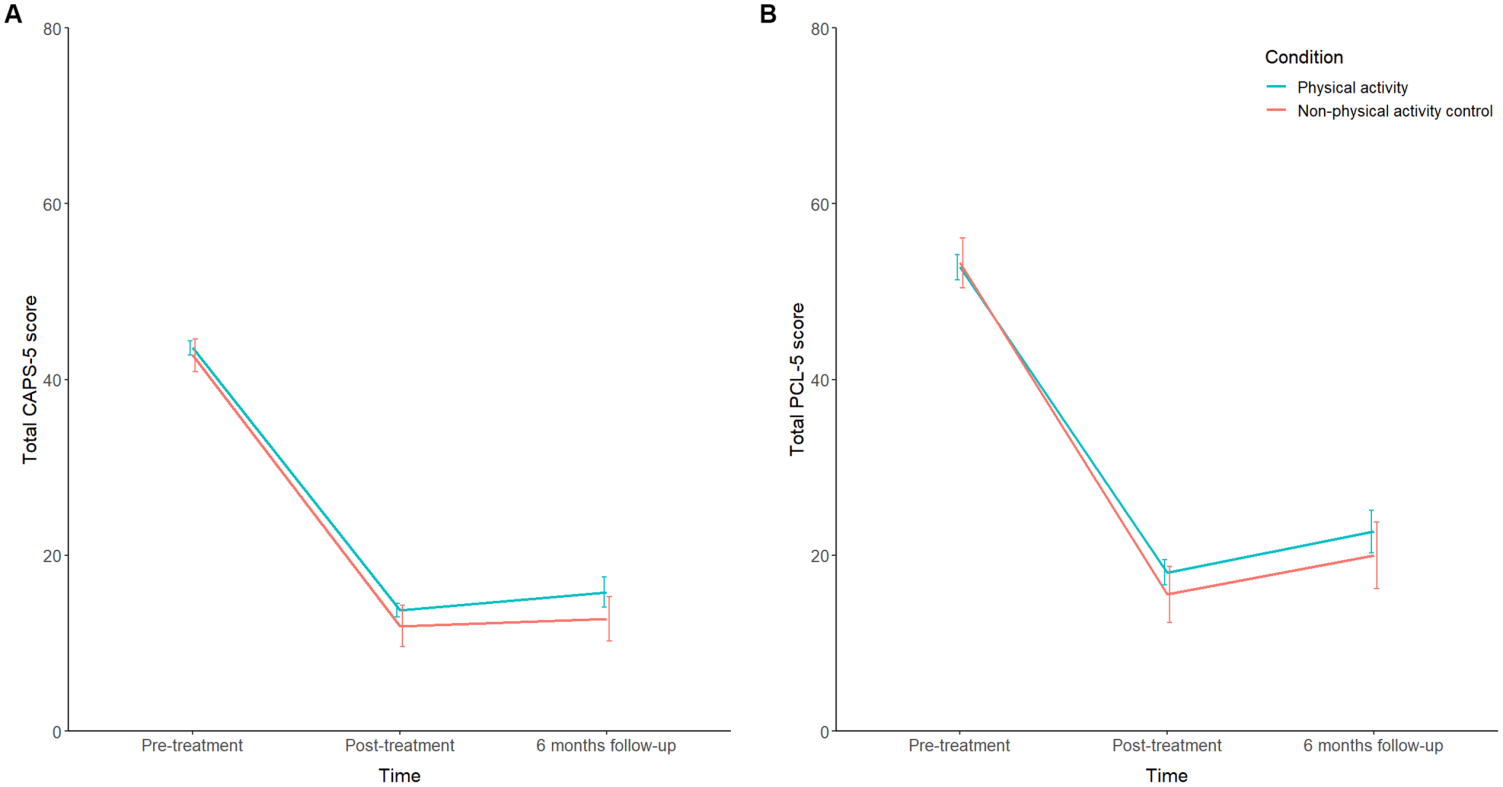
**(A)** Mean CAPS-5 scores over the three time points for both conditions. **(B)** Mean PCL-5 scores over the three time points for both conditions. CAPS, Clinician-Administered PTSD Scale; PCL-5, PTSD Checklist for DSM-5 (both score range 0–80). Error bars represent standard error of the mean (s.e.m.).

A linear mixed model revealed an overall significant decrease in CAPS-5 scores following treatment (*F*[2, 200] = 157.64, *p <* 0.001, *f* = 1.80, Table 2), both from pre-to post-treatment (*t*[200] = −16.70, *p* < 0.001, *d* = −2.28) and from pre-treatment to 6 months follow-up (*t*[200] = −15.16, *p* < 0.001, *d* = −2.20). No significant difference was found between the post-treatment and 6 months follow-up CAPS-5 scores (*F*[1, 200] = 1.72, *p* = 0.191). No significant effect of condition (*F*[1, 117] = 0.55, *p* = 0.459) or condition-by-time interaction effect was observed (*F*[2, 200] = 0.37, *p* = 0.691). Hence, the CAPS-5 scores decreased significantly over time in both conditions (PA: *d*_pre-post_ = −2.11; *d*_pre-fu_ = −1.98; nPA: *d*_pre-post_ = −2.48; *d*_pre-fu_ = −2.46), without significant differences between the two conditions (Figure 2A).

**Table 2:** Model summary final model for the prediction of CAPS-5 and PCL-5 scores for both conditions (*N* = 119^a^).

	CAPS-5			PCL-5		
Parameter	Value	*SE*	*p*-value	Value	*SE*	*p*-value
Intercept^b^	43.59	0.79	<0.001	52.80	1.43	<0.001
Time (versus pre-treatment)
*Post-treatment*	−29.84	1.79	<0.001	−34.74	2.63	<0.001
*6 months follow-up*	−27.78	1.83	<0.001	−30.09	2.94	<0.001
Non-physical activity control (versus physical activity condition)	−0.83	1.11	0.459	0.47	2.01	0.815
Time * condition
Post-treatment * condition	−0.98	2.56	0.701	−2.97	3.75	0.430
6 months follow-up * condition	−2.20	2.61	0.402	−3.19	4.15	0.444

a Total sample size of people in the physical activity (*n*_1_ = 59) and non-physical activity control (*n*_2_ = 60) conditions.

b Reference group (intercept): physical activity condition at pre-treatment.*Note*. CAPS, Clinician-Administered PTSD Scale; PCL-5, PTSD Checklist for DSM-5.

An overall significant decrease in PCL-5 scores following treatment was found (*F*[2, 196] = 87.20, *p <* 0.001, *f* = 1.38), both from pre-to post-treatment (*t*[196] = −13.19, *p <* 0.001, *d* = −1.85), and from pre-treatment to 6 months follow-up (*t*[196] = −10.24, *p <* 0.001, *d* = −1.51; Table 2). A significant increase in PCL-5 scores was observed between post-treatment and 6 months follow-up (*F*[1, 196] = 5.36, *p* = 0.022, *d* = 0.33). No significant effect of condition (*F*[1, 117] = 0.05, *p* = 0.815) or condition-by-time interaction effect was observed (*F*[2, 196] = 0.35, *p* = 0.707). Hence, the PCL-5 scores decreased significantly over time in both conditions (PA: *d*_pre-post_ = −1.70; *d*_pre-fu_ = −1.52; nPA: *d*_pre-post_ = −2.03; *d*_pre-fu_ = −1.51), without significant differences between the two conditions (Figure 2B).

### 3.3. Loss of PTSD and CPTSD diagnoses

Overall, 87 patients (81.3%) lost their PTSD diagnosis post-treatment based on the CAPS-5, 44 patients (80.0%) in the PA condition and 43 patients (82.7%) in the nPA condition (*χ*^2^[1] = 0.13, *p* = 0.721). Furthermore, at 6 months follow-up, a total of 78 patients (80.4%) no longer fulfilled the diagnostic criteria for PTSD, 37 patients (75.5%) in the PA condition and 41 patients (85.4%) in the nPA condition (χ^2^[1] = 1.51, *p* = 0.219).

Furthermore, of the 80 patients (67.2%) who met the diagnostic criteria for CPTSD at baseline, 75 (93.8%) lost their CPTSD diagnosis at post-treatment, 37 of the 40 patients (92.5%) in the PA condition and 38 of the 40 patients (95.0%) in the nPA condition (*χ*^2^[1] = 1.31, *p* = 0.727).

### 3.4. Analysis of secondary outcomes

**Figure 3:**
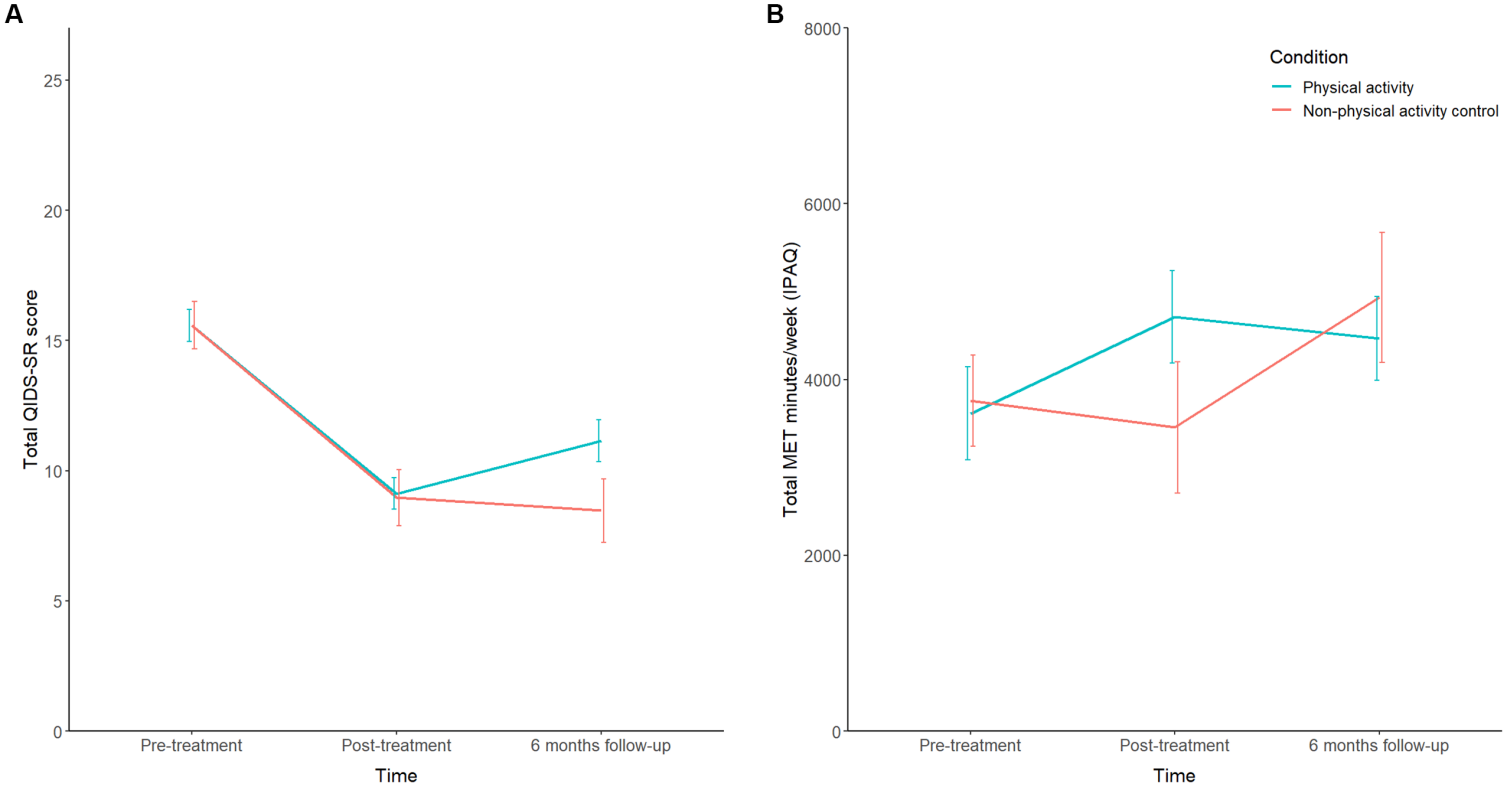
**(A)** Mean QIDS-SR scores over the three time points for both conditions. **(B)** Mean IPAQ-SF scores (MET minutes per week) over the three time points for both conditions. QIDS-SR, Quick Inventory of Depressive Symptomatology Self-Report (score range 0–27); IPAQ-SF, International Physical Activity Questionnaire Short Form; MET minutes = Metabolic Equivalent Task minutes reflecting energy expenditure, with walking, moderate and vigorous intensity being, respectively, 3.3 METs, 4.0 METs, and 8.0 METs (calculated based on IPAQ scoring protocol). Error bars represent standard error of the mean (s.e.m.).

Overall, linear mixed models showed large and significant decreases in sleep problems, depressive symptoms, emotion regulation problems, dissociative symptoms, anxiety sensitivity, general somatic complaints, and CPTSD symptom scores, and significant increases in quality of life scores following treatment (all *F*s > 16.0, *p*s < 0.001, and *f*s > 0.60; Supplementary Table 1). No significant condition-by-time interaction effects for any of these outcomes were found (all *F*s < 2.97, *p*s > 0.053; Supplementary Figures 1–7), except for depressive symptoms (*F*[2,195] = 3.57, *p* = 0.030, *f* = 0.19), which showed a significant increase only in the PA condition from post-treatment to 6 months follow-up (*F*[1,195] = 8.17, *p* = 0.005, *d* = 0.39; Figure 3A). With regard to the physical activity level, no significant increase over time was found (*F*[2,192] = 2.06, *p* = 0.130, *f* = 0.18). Although the conditions presented differing physical activity level patterns over time, this condition-by-time interaction was not significant (*F*[2,192] = 2.53, *p* = 0.082; Figure 3B). However, exploratory post-hoc analysis showed a significant increase in physical activity level between post-treatment and 6 months follow-up in the nPA condition (*F*[1,192] = 6.54, *p* = 0.011, *d* = 0.35).

### 3.5. Treatment preference

At baseline, 71 participants (59.7%) reported a preference for additional physical activity, whereas 48 (40.3%) preferred non-physical creative tasks. After randomization, 51 patients (42.9%) received their preferred treatment condition and 68 (57.1%) did not receive their preference. Whether participants received their preferred treatment condition did not influence the treatment effectiveness (CAPS-5 and PCL-5 decrease) as there were no significant randomized condition x preferred treatment x time interactions (*F*[2,186] = 0.57, *p* = 0.568 and F[1.66, 149.36] = 0.85, *p* = 0.412, respectively).

## 4. Discussion

The present randomized controlled trial is the first to compare the effectiveness of additional physical activity (PA) to a non-physical activity control condition (nPA) within intensive trauma-focused treatment (TFT) in a sample of patients with (Complex) PTSD. PTSD symptoms significantly decreased for both the PA and nPA conditions, with large effect sizes (e.g., CAPS-5 *d_pre-post_* = 2.28), but without significant differences between conditions. Loss of diagnosis was high in both groups. At 6 months follow-up, there was a small relapse in depressive symptoms in the PA condition, while exploratory analyses showed an increase in physical activity levels in the nPA condition. No differences between conditions were found regarding sleep and emotion regulation problems, dissociative symptoms, anxiety sensitivity, quality of life, general somatic complaints, and Complex PTSD symptoms. Treatment condition preference had no influence on treatment effectiveness.

Although several PTSD treatment programmes worldwide incorporate physical activity in their (intensive) TFT formats (Van Woudenberg et al., 2018; Zalta et al., 2018; Zepeda Méndez et al., 2018; Voorendonk et al., 2020; Brynhildsvoll Auren et al., 2022), and a recent qualitative study emphasized that patients report physical activity as an essential part of intensive treatment (Thoresen et al., 2022), our results suggest that physical activity is not a necessary part of intensive TFT for patients with PTSD in comparison to adding non-physical activity control tasks. This finding contrasts with the promising effects of physical activity reported in previous trials and meta-analyses (Rosenbaum et al., 2015b; Björkman and Ekblom, 2021; Davis et al., 2021; Ramos-Sanchez et al., 2021; Bryant et al., 2022). However, these findings are in agreement with other recent studies showing null findings or smaller effect sizes of physical activity on PTSD or anxiety symptoms (Whitworth et al., 2019b; Nordbrandt et al., 2020; Bichler et al., 2022; Philippot et al., 2022; Young-McCaughan et al., 2022), and are somewhat in line with our previous study showing that increases in cardiorespiratory fitness were not significantly related to decreases in PTSD symptoms following intensive TFT (Voorendonk et al., 2019).

Based on the current overall large effect sizes (e.g., CAPS-*5 d_pre-post_* = 2.28) and loss of PTSD diagnostic status rates at post-treatment (81.3%) and follow-up (80.4%) for both conditions, it is possible that the current trial was prone to a ceiling effect. The intensive TFT, consisting of two evidence-based psychological treatments, might have already generated such a large effect that it left no room to augment the treatment any further, regardless of condition. This is supported by the large effect sizes reported in previous research on intensive TFT, also without additional physical activity (*d*s ≥ 1.04; Ehlers et al., 2014; Foa et al., 2018; Hendriks et al., 2018; Oprel et al., 2021; Dell et al., 2022). Although pragmatically difficult, our trial should ideally have included a third control condition with intensive TFT without any additional physical or non-physical activities to draw conclusions on the augmentation effects. Regardless, from a clinical point of view, the relatively large effect sizes, loss of diagnostic status rates and low drop-out rates in both groups in our trial are very promising treatment outcome results for patients with (Complex) PTSD in general.

Previously reported augmentation effects of physical activity on TFT might be explained by non-specific factors, such as receiving more attention, experiencing social support, being distracted, working memory taxation, or behavioral activation (Asmundson et al., 2013; Etherton and Farley, 2020; Bichler et al., 2022). More specifically, the group participation in our trial, in both the PA and nPA condition, could have been a factor of importance. Social support from and interaction with other group members cause a reduction in social withdrawal which could have beneficial effects on PTSD symptoms (e.g., Asmundson et al., 2013; Bichler et al., 2022). The same could hold true for receiving additional attention and social support from the physical and non-physical activity instructors. In line with our current findings, previous studies that incorporated equal contact control groups (individual or group) revealed smaller effect sizes or null-findings of physical activity on PTSD or anxiety symptoms as stand-alone treatment option or as an add-on to psychological therapies (Whitworth et al., 2019a,b; Nordbrandt et al., 2020; Bichler et al., 2022; Philippot et al., 2022; Young-McCaughan et al., 2022). Moreover, since the physical activities were 85% performed outside, in a natural environment, it is important to mention that the time spent outdoors could have also been of influence. For instance, physical activities in natural environments (‘green exercise’) are significantly associated with improved mental health compared to urban environments (Bowler et al., 2010; Gidlow et al., 2016; Zijlema et al., 2018). More specifically, it has been shown that the more time veterans with PTSD spent outdoors, the lower their PTSD symptom severity (Bettmann et al., 2021). Nevertheless, since the non-physical activities in our trial were 10% performed outside, and we did not find a significant difference between the two groups, these beneficial effects of being outside could not have had a large effect on our findings.

There were overall significant reductions in clinician-administered PTSD symptoms from pre-to post-treatment, which remained stable at 6 months follow-up (CAPS-5). However, on the self-report measure (PCL-5) the overall decreases in PTSD symptoms after treatment showed a slight relapse from post-treatment to 6 months follow-up. This difference could be explained by observations from research showing that the severity of PTSD symptoms measured with self-report are significantly higher than when measured using the clinician-administered PTSD scale (Kramer et al., 2023). This overreporting on self-report measures could be caused by patients having more difficulty responding differentially to similar symptoms (e.g., nightmares and sleeping problems), while clinicians might be better able to differentiate between these symptoms, and not score them twice (Kramer et al., 2023). Yet, the fact that participants still showed large improvements after 6 months in comparison to pre-treatment, both on the clinician-administered and the self-report measure, remains an encouraging finding for clinical practice. Moreover, the relapse of depressive symptoms in the PA condition and the increase in physical activity level in the nPA condition at 6 months follow-up, were unexpected findings in favor of the control condition. The latter increase might be explained by the fact that participants in the nPA condition were restricted in movement during treatment, which may have resulted in compensatory physical activity behavior after treatment and, consequently, protected the control condition from a relapse in depressive symptoms (Schuch et al., 2016).

### 4.1. Strengths and limitations

Important strengths of our study are (1) the inclusion of a non-physical activity control condition, (2) long-term assessment at 6 months follow-up, and (3) the heterogeneous sample of patients with PTSD, reflected by their exposure to a wide variety of multiple traumas and high comorbidity rates, which optimizes the generalization of our findings. The trial had relatively low dropout rates during treatment (5.9%) (Vancampfort et al., 2021) and low attrition rates, even at 6 months follow-up (18.4%) (Schottenbauer et al., 2008). There was good adherence to the treatment protocols, assessed by objective and subjective measures, and both clinician-administered and self-reported measures of PTSD symptoms were incorporated.

Our study also has some limitations. First, it is possible that the controlled (creative) tasks in the current nPA condition had beneficial effects, resulting in a lack of difference between the two conditions. Preliminary evidence shows that even (mandala) coloring as a stand-alone activity can reduce PTSD symptoms in veterans (Rodak et al., 2018). Moreover, Philippot et al. (2022), who included a similar creative control condition, found that the physical activity condition was as beneficial as the creative task control intervention on anxiety symptoms. However, evidence for the effectiveness of creative therapy in patients with PTSD is scarce (Baker et al., 2017). Again, the common non-specific elements of non-physical (creative) group activities, such as providing social support, receiving attention, offering distraction from rumination and a form of behavioral activation, could have resulted in the lack of difference between the conditions. Unfortunately, these non-specific factors, such as social support, were not directly measured in our randomized controlled trial, neither did we have an intensive TFT-only group to assess these effects. Second, we pragmatically could not incorporate assessors blinded to treatment conditions, which could have influenced the study outcomes. However, we did not find any significant differences between the conditions on both the clinician-administered and self-report measures, making it less likely that an observer bias may have influenced the results. Third, there was no information on the received care during the 6 months following treatment, so the follow-up results should be treated with caution. Fourth, the PA condition may not have been provided at optimal intensity, duration and sequence. The World Health Organization guidelines recommend on average 150–300 minutes of moderately intensive, or 75–150 minutes of vigorous intensive aerobic physical activities throughout the week to prevent diseases and maintain physical health (World Health Organization, 2010; Bull et al., 2020). Based on their heart rate monitors, participants in our trial engaged in around 288 minutes of moderate intensity per week, which falls in the range of the WHO. Moreover, the brief duration of two weeks, instead of longer durations in previous research (e.g., >12 weeks), and variable sequences, could have influenced the results. In the context of intensive and brief TFT for patients with PTSD, future studies should explore different intensities, durations, and sequences of physical activity and/or exercise (Björkman and Ekblom, 2021; Davis et al., 2021; Ramos-Sanchez et al., 2021; Voorendonk et al., 2021; Crombie et al., 2023). Fifth, our physical activity intervention consisted of multiple components, including aerobic, resistance and competitions/games, making it difficult to disentangle their unique effects. However, it is likely that our findings are more generalizable to clinical practice where PTSD treatment programmes worldwide often incorporate these multi-component forms of physical activity into their intensive TFT formats (Van Woudenberg et al., 2018; Zalta et al., 2018; Zepeda Méndez et al., 2018; Voorendonk et al., 2020; Brynhildsvoll Auren et al., 2022; Thoresen et al., 2022). Moreover, there is still a lot unknown about the optimal type and level of physical activity for patients with PTSD. Sixth, this was a single-institution trial, with a sample of predominantly Dutch females, so one may not be able to infer to a broader population of men, especially since emerging evidence supports gender differences in the relationship between physical activity and PTSD symptoms (Whitworth et al., 2017). Finally, of those assessed for eligibility a high percentage declined to participate (56%), which may have resulted in a biased sample.

### 4.2. Conclusion

This randomized controlled trial showed that intensive TFT, either with additional physical or non-physical activities, was very effective for (Complex) PTSD, reflected by large effect sizes, significant loss of diagnostic status, and very low dropout rates in both groups. Replication and additional research on the impact of physical activity on PTSD treatment is recommended.

## Data availability statement

The data presented in this article are not readily available because of privacy restrictions but are available from the corresponding author upon reasonable request. Requests to access the data should be directed to e.voorendonk@psytrec.nl.

## Ethics statement

The studies involving human participants were reviewed and approved by the Medical Ethics Review Committee of the VU University Medical Center (VUmc: IRB00002991, FWA0001 7598; reference number: 2019.574 – NL70812.029.19). The patients/participants provided their written informed consent to participate in this study.

## Author contributions

EV had full access to all the data in the study and takes responsibility for the integrity of the data and the accuracy of the data analysis. EV, AM, AJ, SS, and MT: concept and design. EV, AM, AJ, and SS: acquisition, analysis, or interpretation of data, drafting of the manuscript, and obtained funding. EV, AM, AJ, SS, MT, and EH: critical revision of the manuscript for important intellectual content. EV and EH: Statistical analysis. AM and AJ: Administrative, technical, or material support and supervision. All authors contributed to the article and approved the submitted version.

## Funding

This work was supported in part by the EMDR Research Foundation, Vereniging EMDR Nederland (VEN), for the acquisition of heart rate monitors and materials. The EMDR Research Foundation had no role in any part of the study (design, data collection, management, analysis, interpretation, review).

## Conflict of interest

AM receives income from published book chapters on PTSD and from the training of postdoctoral professionals in prolonged exposure. AJ receives income from published books on EMDR therapy and from the training of postdoctoral professionals using this method. AM, AJ, and EV are employed at PSYTREC.The remaining authors declare that the research was conducted in the absence of any commercial or financial relationships that could be construed as a potential conflict of interest.

## Publisher’s note

All claims expressed in this article are solely those of the authors and do not necessarily represent those of their affiliated organizations, or those of the publisher, the editors and the reviewers. Any product that may be evaluated in this article, or claim that may be made by its manufacturer, is not guaranteed or endorsed by the publisher.
