# Supplementary material for: Adding physical activity to intensive trauma-focused treatment for post-traumatic stress disorder: results of a randomized controlled trial
Source: Front Psychol. 2023 Jul 20;14:1215250. doi: 10.3389/fpsyg.2023.1215250 (PMC10400339; doi:10.3389/fpsyg.2023.1215250)
Supplement: Supplementary file 1 [file Table_1.pdf]

Supplementary Table 1.

Primary and secondary mean outcome scores (and standard deviations) during pre-treatment, post-treatment and at 6 months follow-up for the physical activity (PA; *n* = 59) and non-physical activity control condition (nPA; *n* = 60).

	PA			nPA		
Outcome (mean (*SD*) scores)	Pre	Post	6 months follow-up	Pre	Post	6 months follow-up
Primary outcomes						
CAPS-5	43.59 (6.34)	13.69 (13.13)	14.80 (14.07)	42.77 (5.79)	11.94 (12.00)	12.44 (11.81)
PCL-5	52.80 (11.92)	17.98 (18.84)	22.15 (19.64)	53.27 (9.89)	15.33 (16.88)	19.23 (20.74)
Secondary outcomes						
ISI	18.44 (5.14)	10.20 (7.11)	12.28 (6.80)	18.28 (5.27)	11.04 (6.29)	9.87 (7.34)
QIDS-SR	15.58 (4.88)	9.07 (6.20)	10.89 (6.60)	15.58 (4.44)	8.96 (5.62)	8.09 (6.44)
DERS	117.59 (24.05)	85.65 (24.39)	89.78 (28.39)	116.32 (23.40)	83.23 (26.99)	83.17 (31.76)
DES-II	25.53 (17.26)	12.94 (14.67)	15.48 (18.40)	25.18 (14.13)	13.67 (14.15)	14.07 (15.09)
ASI	26.48 (12.61)	13.70 (12.28)	16.52 (14.10)	21.13 (10.28)	10.90 (9.40)	11.53 (10.89)
MANSA	47.07 (8.95)	55.47 (10.54)	53.48 (11.17)	46.70 (8.83)	55.90 (10.85)	56.11 (12.20)
IPAQ-SF						
MET mins/week	3614.47 (4304.00)	4765.38 (3767.06)	4521.45 (3577.31)	3759.27 (3809.48)	3518.98 (3253.91)	5085.65 (3693.73)
Sitting mins/day	445.68 (185.52)	340.74 (182.23)	315.33 (146.47)	421.30 (217.78)	354.06 (223.42)	350.20 (203.17)
SCL-90	33.02 (9.66)	22.13 (10.02)	25.04 (10.35)	30.98 (8.06)	20.85 (6.99)	21.33 (7.40)
ITQ	33.47 (7.88)	11.96 (12.60)	16.70 (13.16)	33.20 (6.60)	11.46 (11.19)	13.16 (12.59)

Note. CAPS-5 = Clinician-Administered PTSD Scale for DSM-5; PCL-5 = PTSD Checklist for DSM-5; ISI = Insomnia Severity Index; QIDS-SR = Quick Inventory of Depressive Symptomatology Self-Report; DERS = Difficulties in Emotion Regulation Scale; DES-II = Dissociative Experiences Scale-II; ASI = Anxiety Sensitivity Index; MANSA = Manchester Short Assessment of Quality of Life; IPAQ-SF = International Physical Activity Questionnaire Short Form; MET minutes = Metabolic Equivalent Task minutes reflecting energy expenditure, with walking, moderate and vigorous intensity being respectively 3.3 METs, 4.0 METs, and 8.0 METs (calculated based on IPAQ scoring protocol); SCL-90 = Symptom Check List-90, somatization subscale; ITQ = International Trauma Questionnaire.
