# Supplementary material for: Adding physical activity to intensive trauma-focused treatment for post-traumatic stress disorder: results of a randomized controlled trial
Source: Front Psychol. 2023 Jul 20;14:1215250. doi: 10.3389/fpsyg.2023.1215250 (PMC10400339; doi:10.3389/fpsyg.2023.1215250)
Supplement: Supplementary file 2 [file Image_1.pdf]

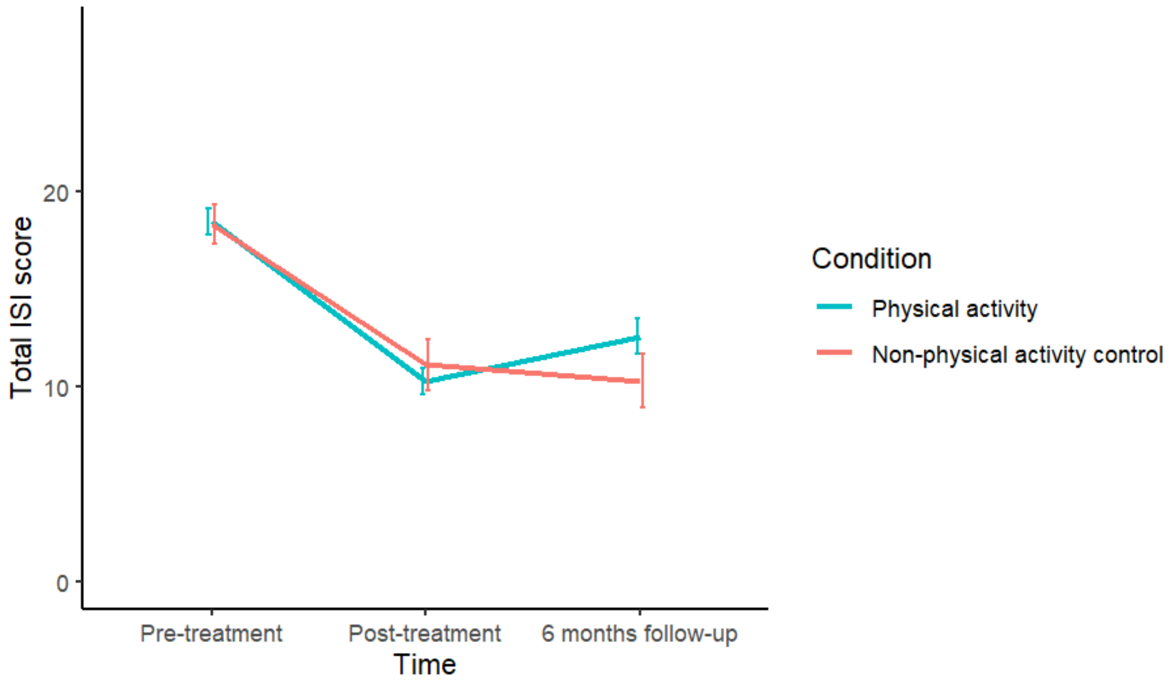

Supplementary Figure 1. Mean ISI scores over the three time points for both conditions. *Note*. ISI = Insomnia Severity Index (score range 0-28). Error bars represent standard error of the mean (s.e.m.).

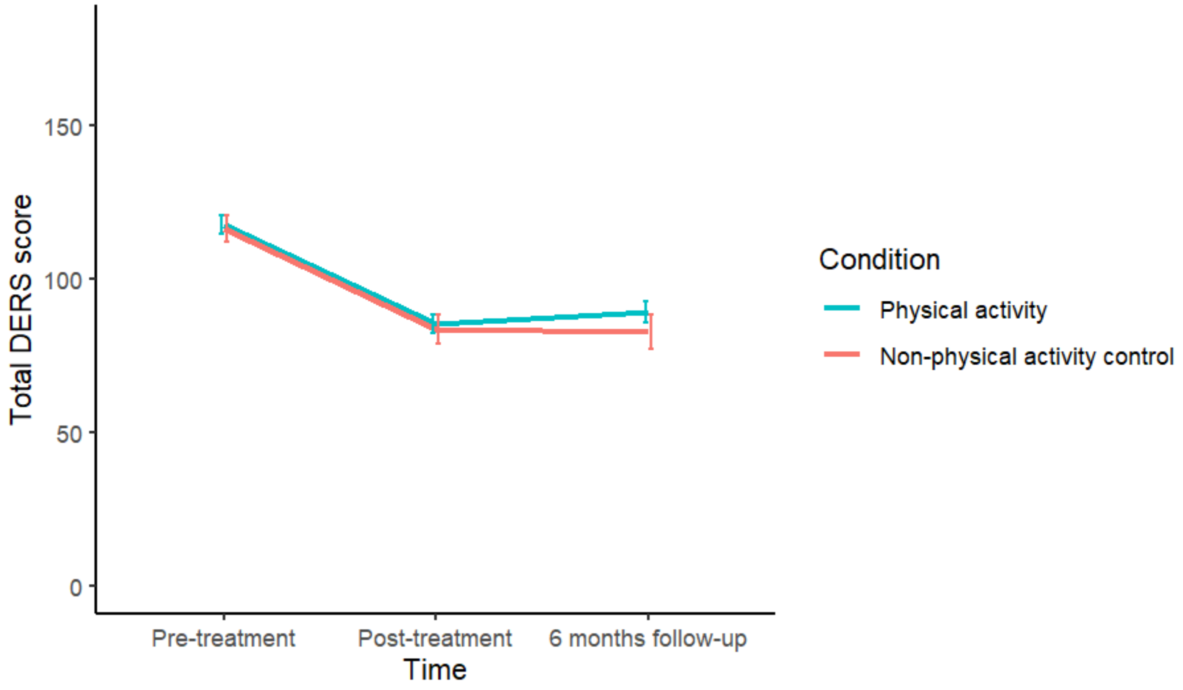

Supplementary Figure 2. Mean DERS scores over the three time points for both conditions.

Note. DERS = Difficulties in Emotion Regulation Scale (score range 36-180). Error bars represent standard error of the mean (s.e.m.).

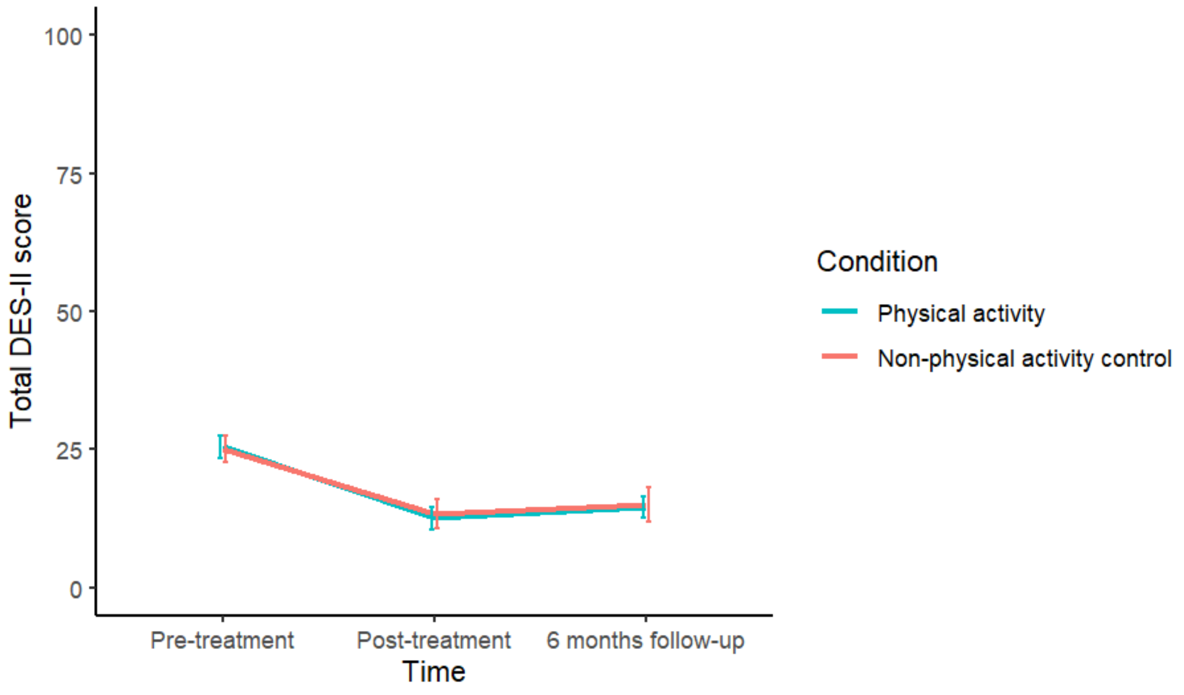

Supplementary Figure 3. Mean DES-II scores (percentages) over the three time points for both conditions. *Note.* DES-II = Dissociative Experiences Scale-II (score range 0-100). Error bars represent standard error of the mean (s.e.m.).

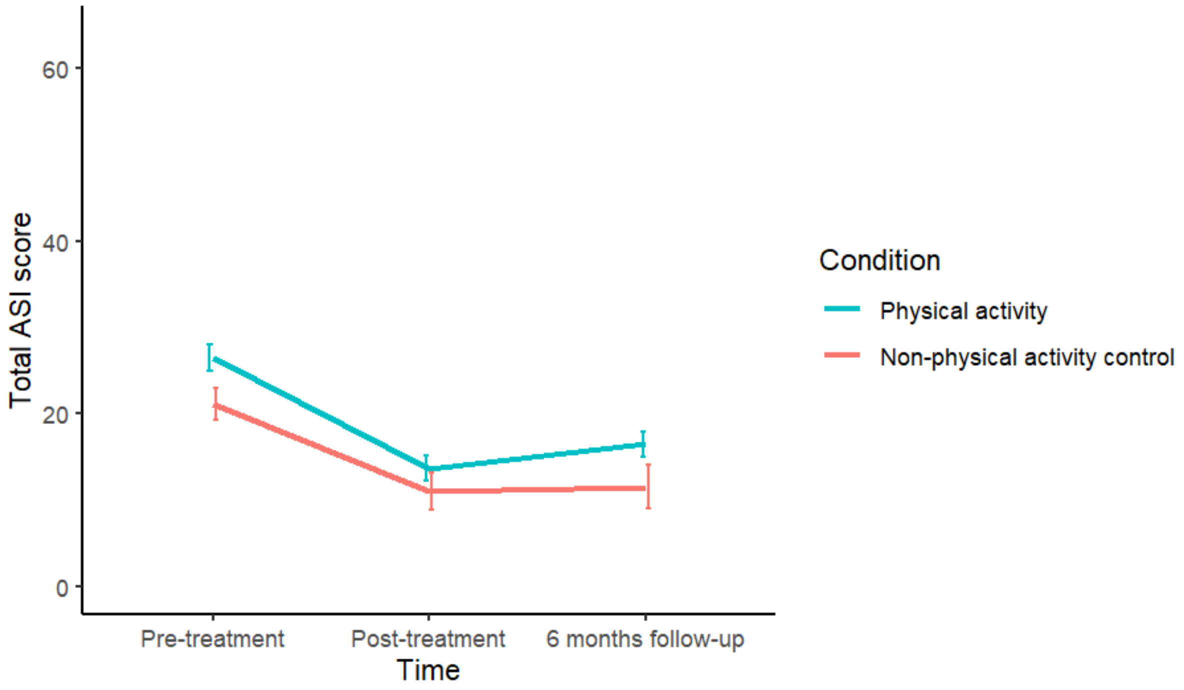

Supplementary Figure 4. Mean ASI scores over the three time points for both conditions. *Note.* ASI = Anxiety Sensitivity Index (score range 0-64). Error bars represent standard error of the mean (s.e.m.).

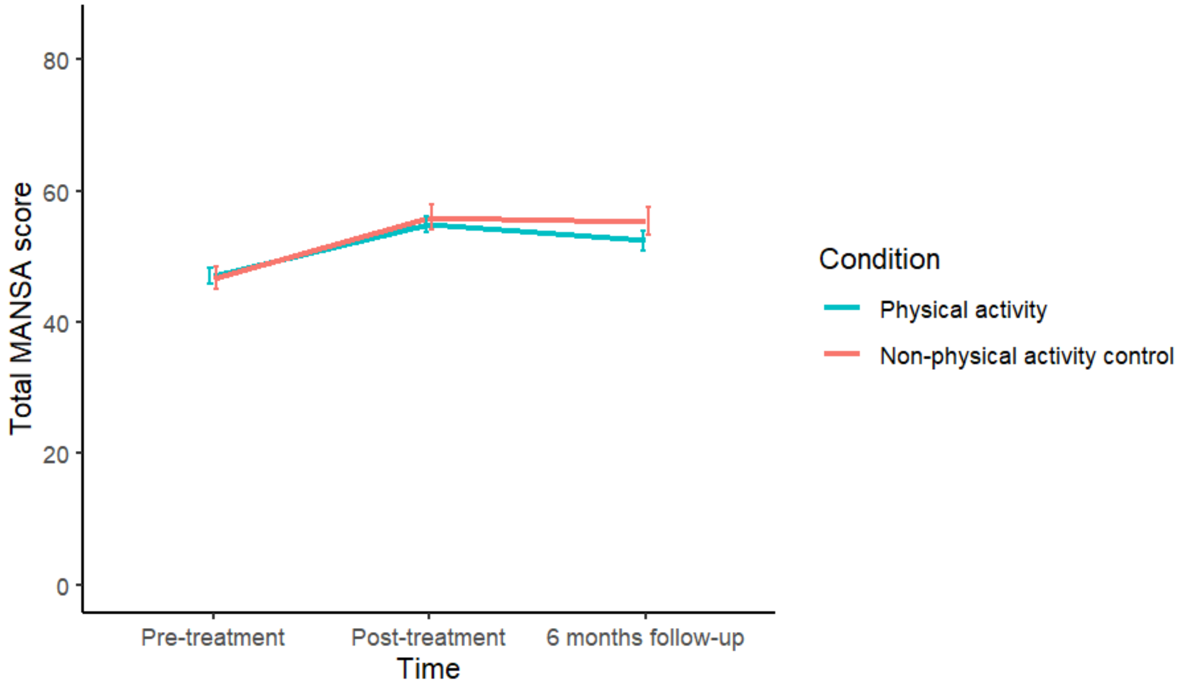

Supplementary Figure 5. Mean MANSA scores over the three time points for both conditions.

Note. MANSA = Manchester Short Assessment of Quality of Life (score range 12-84). Error bars represent standard error of the mean (s.e.m.).

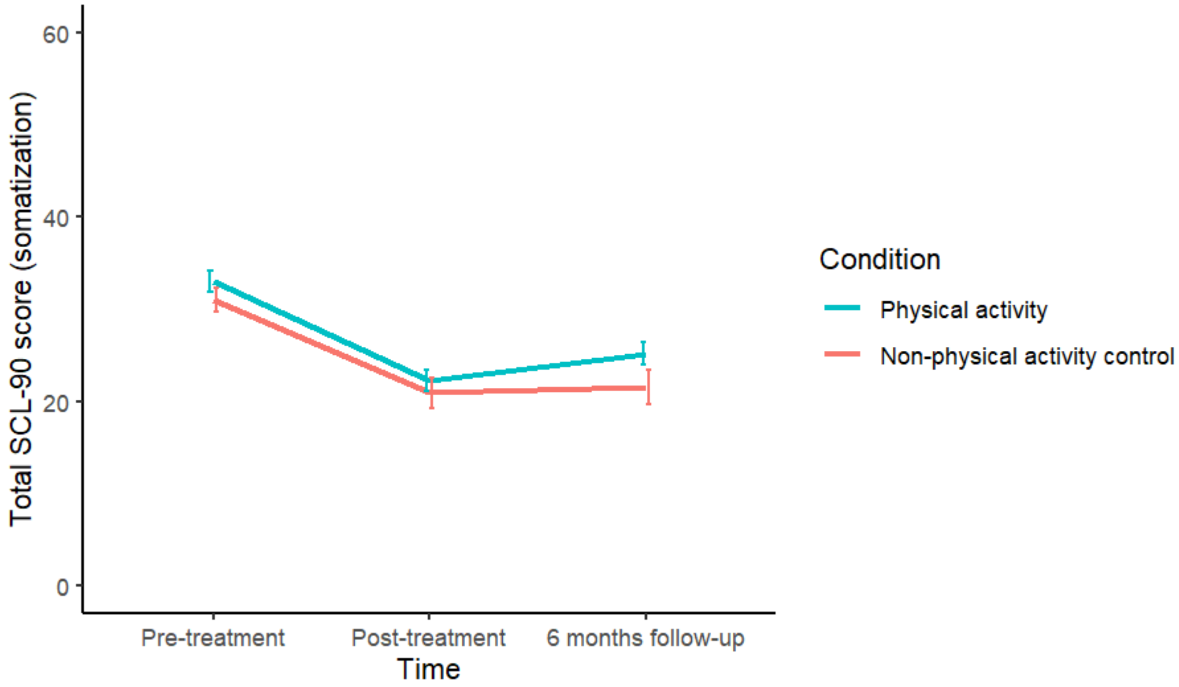

Supplementary Figure 6. Mean SCL-90 (somatization subscale) scores over the three time points for both conditions. *Note.* SCL-90 = Symptom Check List-90, somatization subscale (score range 12-60). Error bars represent standard error of the mean (s.e.m.).

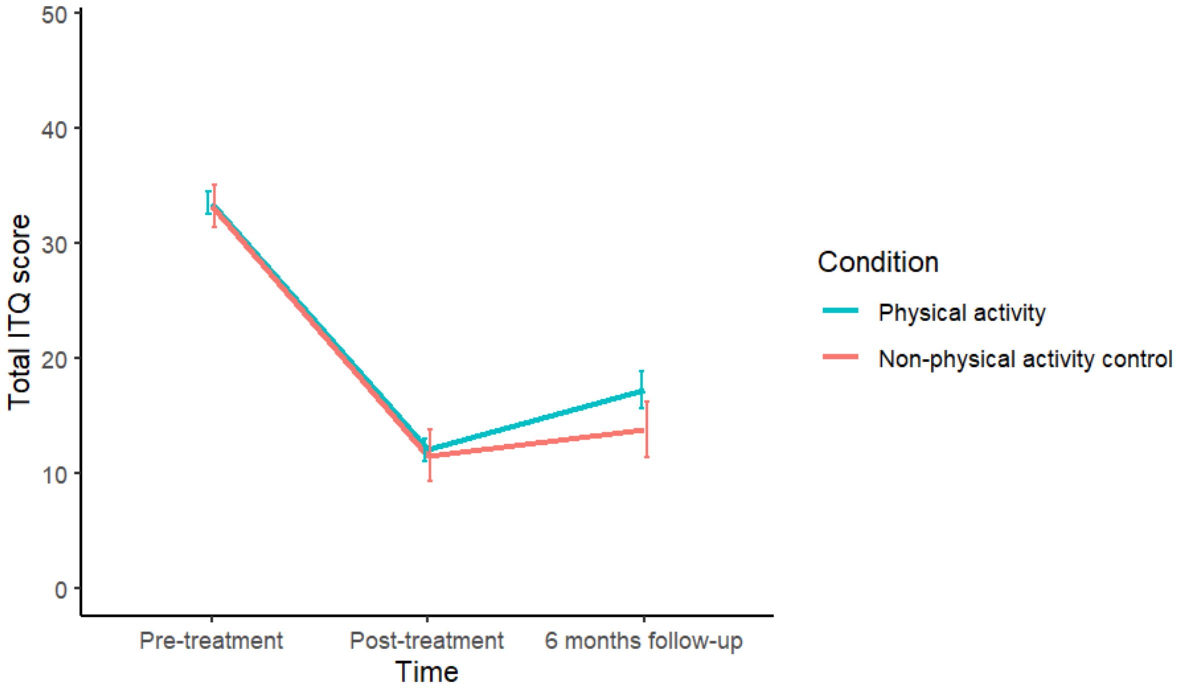

Supplementary Figure 7. Mean ITQ scores over the three time points for both conditions.

Note. ITQ = International Trauma Questionnaire (score range 0-48). Error bars represent standard error of the mean (s.e.m.).
