# Supplementary material for: Adding physical activity to intensive trauma-focused treatment for post-traumatic stress disorder: results of a randomized controlled trial
Source: Front Psychol. 2023 Jul 20;14:1215250. doi: 10.3389/fpsyg.2023.1215250 (PMC10400339; doi:10.3389/fpsyg.2023.1215250)
Supplement: Supplementary file 3 [file Image_2.pdf]

Supplementary results

Sensitivity analysis of primary outcome measures (CAPS-5 and PCL-5) with participant without PTSD diagnosis at baseline included.

A linear mixed model revealed an overall significant decrease in CAPS-5 scores following treatment (F[2,202] = 146.93, $p < 0.001$, $f = 1.74$), both from pre- to post-treatment (t[202] = -16.09, $p < 0.001$, $d = -2.20$) and from pre-treatment to 6 months follow-up (t[202] = -14.87, $p < 0.001$, $d = -2.14$). No significant difference was found between post-treatment and 6 months follow-up CAPS-5 scores (F[1,202] = 1.54, $p = 0.216$). No significant effect of condition (F[1,118] = 0.20, $p = 0.653$), or condition-by-time interaction effect was found (F[2,202] = 0.52, $p = 0.598$).

An overall significant decrease in PCL-5 scores following treatment was found (F[2,198] = 71.66, $p < 0.001$, $f = 1.27$), both from pre- to post-treatment (t[198] = -11.97, $p < 0.001$, $d = -1.69$) and from pre-treatment to 6 months follow-up (t[202] = -9.25, p < .0.001, $d = -1.37$). A significant increase in PCL-5 scores was observed between post-treatment and 6 months follow-up (F[1,198] = 5.80, $p = 0.017$, $d = 0.34$). No significant effect of condition (F[1,118] = 0.25, $p = 0.618$), or condition-by-time interaction effect was found (F[2,198] = 0.69, $p = 0.501$).
